# Printable Highly Stable and Superfast Humidity Sensor Based on Two Dimensional Molybdenum Diselenide

**DOI:** 10.1038/s41598-020-62397-x

**Published:** 2020-03-26

**Authors:** Muhammad Awais, Muhammad Umair Khan, Arshad Hassan, Jinho Bae, Tahseen Elahi Chattha

**Affiliations:** 1 National University of Computer & Emerging Sciences (NUCES), Islamabad, Pakistan; 2 Department of Ocean System Engineering, Jeju National University, 102 Jejudaehakro, Jeju, 63243 Republic of Korea

**Keywords:** Engineering, Materials science

## Abstract

Transition metal dichalcogenides (TMDCs) are promising materials for sensing applications, due to their exceptional high performance in nano-electronics. Inherentely, the chemical and thermal responses of TMDCs are highly stable, hence, they pave way for real time sensor applications. This article proposes inceptively a stable and superfast humidity sensor using two-dimensional (2D) Molybdenum diselenide (MoSe_2_) through printed technlogies. The 2D MoSe_2_ ink is synthesized through wet grinding to achieve few-layered nano-flakes. Inter digital electrodes (IDEs) are fabricated via screen-printing on Polyethylene terephthalate (PET) substrate and thin film of MoSe_2_ nano-flakes is fabricated through spin coating. The impedance and capacitance response are recorded at 1 kHz between temperature levels ranging from 20–30 °C. The impedance and capacitance hysteresis results are recorded <1.98% and <2.36%, respectively, ensuring very good repeatability during humidification and dehumidification. The stability of impedance and capacitance response are recorded with maximum error rate of ~ 0.162% and ~ 0.183%, respectively. The proposed sensor shows fast impedance response time (*T*_*res*_) of ~ 0.96 s, and recovery time (*T*_*rec*_) of ~ 1.03 s, which has *T*_*res*_ of ~ 1.87 s, and *T*_*rec*_ of ~ 2.13 s for capacitance. It is aimed to develop a high performance and stable humidity sensor for various monitoring applications.

## Introduction

Two-dimensional (2D) materials exhibit exceptional physical properties when charge and heat are restricted to a planar layer^1^. Moreover, higher surface to volume ratio of 2D materials favor easy fabrication of sensing layers and smaller size of the electronic devices^2^. Discovery of mechanically exfoliated graphene in 2004^3^, offered new ground to nanotechnology having advantages like high carrier mobility^4^, transparency, and flexibility^5^. Carbon atoms are arranged in a honeycomb lattice structure in 2D graphene^1^. However, the approximate zero band gap of the graphene obstructs its utility in electronic sensing devices owing to low on/off ratio. Various 2D materials have been reported as humidity sensors, however, these have long response and recovery times equivalent to ≈10 sec^6–10^. which restricts their usage as humidity sensors in real time monitoring and health services^11^. In recent years, active research is under consideration for development of layered structures of transition metal dichalcogenides (TMDCs), as a low-cost, fast, directly printable 2D semiconductor material. Two-dimensional TMDCs, too, share a high carrier mobility of 500 cm^2^/Vs^12^, and are being utilized in gas^13^, temperature^14^, electronic^15–22^, and optoelectronics^23–27^ sensing applications because of their exceptional properties^23,28^. General formula for TMDC is given as MX_2_ where M can be Sn, W, Mo, V etc., while X can be S, Se, Te etc.

In this work, Molybdenum diselenide (MoSe_2_) is chosen due to its exceptional humidity sensing capabilities^12^, and its higher electrical conductivity. Molybdenum diselenide offers unchanged wear rate under humid conditions even at high temperatures of 350 °C^29^. The Electron Affinity (EA) of 4.42 and Work Function (*φ*) of 5.20 were calculated for MoSe_2_, respectively^30,31^. These results emphasize that electron extraction is much easier for MoSe_2_ as compare to other members of the same family^30^. MoSe_2_ has a higher tendency to donate an electron and interact with hydrogen molecules. MoSe_2_ presents higher electrical conductivity of 10^−3^ Sm^−1^ due to Selenium’s metallic nature^32^. MoSe_2_ has a density of 6.96 g/cm^3^, with a layer thickness of ≈6–7 Å, and ≈2.49 Å and ≈3.29 Å Mo–Se and Se–Se bond lengths, respectively^33–35^. Energy band gap of TMDCs is greater than 1.0 eV^3^, and greater than graphene and its variants. Single layer MoSe_2_ exhibits an energy band gap of 1.55 eV^36–38^. Thus, TMDCs require lesser power for operation than the pervoskites which display a higher energy band gap^39^. MoSe_2_ is catalytically active in hydrogen adsorption^30^ with 100% Hydrogen interactions at edges, and it has a very low edge binding energy of −13.1 meV/f.u. Hydrogen binding energy for Mo–H and Se-H bonds calculated is −32.3 meV/f.u, −13.1 meV/f.u respectively, for MoSe_2_^30^. This makes MoSe_2_ suitable for stable and superfast humidity sensing material.

In the present work, a humidity sensor based on synthesized nano-flakes of MoSe_2_ is demonstrated. The nano-flakes in the synthesized ink have the higher surface roughness contributing to excessive surface area, and utilizing these flakes gave superfast response (~0.96 s) and recovery time (~1.03 s). Fabrication of IDEs of the proposed sensor was performed, with screen-printing technique, while spin coating technique was utilized for printing MoSe_2_ sensing layer. Optimization of the sensor design was achieved by simulation of three sensors with IDEs spacing of 300 µm, 200 µm, and 100 µm for sensor 1, sensor 2, and sensor 3, respectively. Simulation results suggested that size and spacing of electrodes at 100 µm gives the best performance in terms of lowest impedance and highest capacitance because it is desirable for detection in low RH and practical measurement perspective^40,41^. These results were in accordance with the smaller width and smaller spacing criteria^42^. The screen-printing through mask, restricted the resolution of fabrication and optimization of the sensor at 100 µm spacing and width of IDEs, hence the sensor was designed in COMSOL in accordance with the printing limitations as discussed in Section 2 of this paper and sizing optimization is discussed in Supplementary information file. In terms of stability, this work presents the temperature dependence of MoSe_2_ on different humidity levels. The presented MoSe_2_ based humidity sensor is suitable for a mass production as all fabrication steps are compatible with all printed electronic approaches.

## Materials and Methods

### Inter digitated electrodes design and fabrication

**Figure 1:**
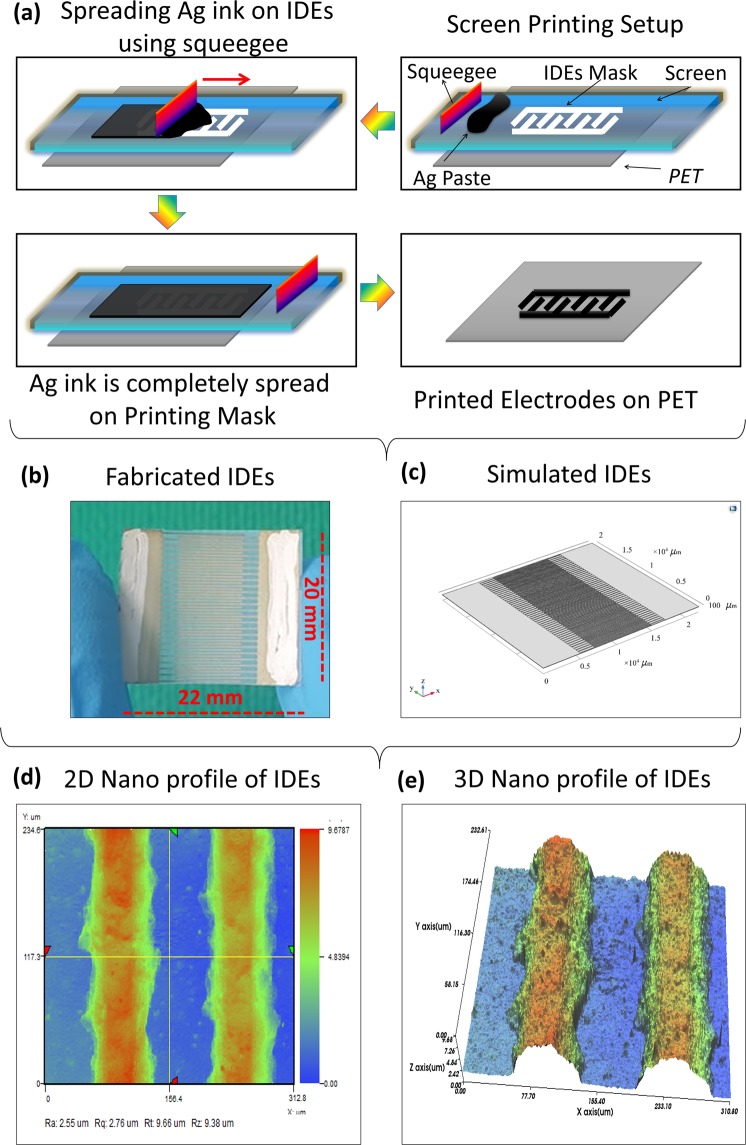
Printing process and Nano-profiles. (**a**) Screen Printing of Ag ink to form IDEs on PET substrate. (**b**) Fabricated sensor. (**c**) Simulated sensor. (**d**) 2D Nano profile of IDEs. (**e**) 3D Nano profile of IDEs.

The Inter Digital Electrodes (IDEs) were fabricated using screen-printer Automax System Engineering AMX-1240M as shown in Fig. 1a. Initially, PET substrate was placed on printing platform, IDEs mask screen was fixed in screen-printer and screen-printing Silver (Ag) ink was placed on printing mask. The squeegee was used to spread ink on printing mask. After complete spreading of Ag ink on printing mask, IDEs were cured at 120 °C. Each electrode consisted of two parts, first a plate of measurement 20 mm × 5 mm length and width respectively with a thickness of 10 µm, second the electrode fingers of 10 mm × 100 µm length and width, respectively, with a thickness of 10 µm. A constant finger spacing of 100 µm was kept between IDEs. The overall size of sensor was 20 mm × 22 mm with a spacer of 2 mm between positive and negative electrode ends. Fabricated IDEs are shown in Fig. 1b. In addition, the IDEs were designed in computer software platform COMSOL Multiphysics 5.3a to verify the practical results with simulated results as shown in Fig. 1c.

### MoSe_2_ ink preparation

Molybdenum (IV) Selenide (MoSe_2_) ~ 325 mesh with 99.9% trace metals basis, N-Methyl-2-Pyrrolidone (NMP) were bought from Sigma Aldrich, South Korea. The screen-printing Ag ink TEC-PA-051LV with viscosity 155,000 ± 15,000 cps, density ~ 2.8 ± 0.2 g/cm^3^, and metal content ~ 70 ± 2 wt% was purchased from InkTec. The 100 µm thick PET substrate was purchased from AgIC paper. MoSe_2_ ink was synthesized via wet grinding assisted co-solvent sonication followed by mechanical shaking, bath sonication, probe sonication and centrifugation a shown in Fig. S6 of Supplementary Information. MoSe_2_ ultra-fine powder was ground in mortar and pester for 8 h using NMP. After grinding, gel like mixture was placed on heater to dry at 110 °C for 1 h. The dried 7 mg mL^−1^ MoSe_2_ powder was mixed in NMP and placed on magnetic stirrer for 24 h at 1,200 rpm. Further, MoSe_2_ solution was probe sonicated for 1 h with on pulse of 1 s and off pulse of 3 s at probe frequency ~ 19.7 kHz and bath sonicated for 30 min. The 2/3 portion of MoSe_2_ solution was centrifuged at 6,000 rpm for 20 min and supernatant was obtained by decantation. The MoSe_2_ nano-flakes film was fabricated using spin coater at 300 rpm for 10 s ramp and 4,000 rpm for 60 s.

### Characterization

**Figure 2:**
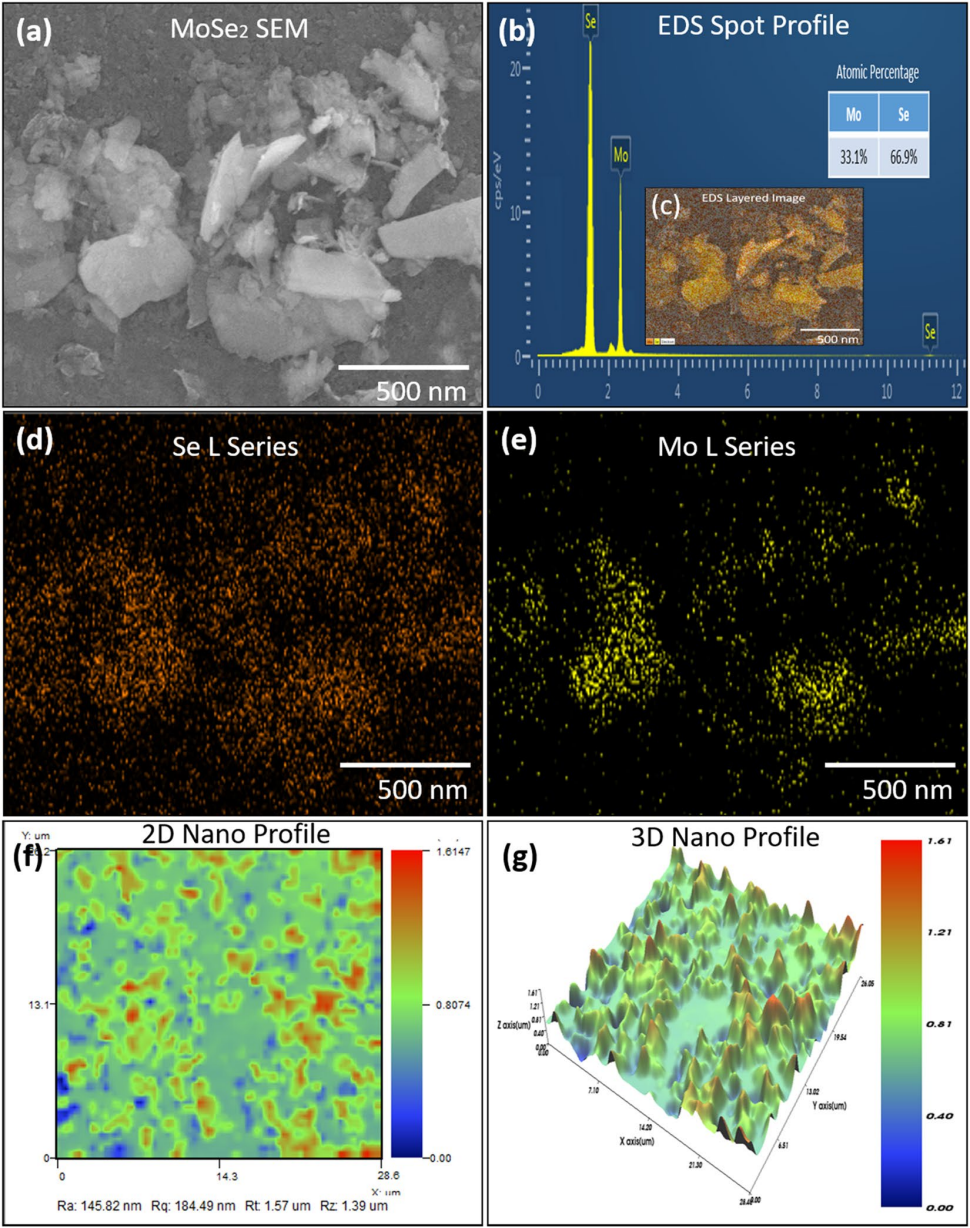
(**a**) MoSe_2_ nano-flakes SEM image. (**b**) EDS spot profile and EDS mapped image and (**c**) EDS layered image. (**d**) Se L series and (**e**) Mo L series. (**f**) 2D Nano profile image showing surface roughness and (**g**) 3D Nano profile showing height profile of MoSe_2_.

The surface morphology of MoSe_2_ was analyzed with scanning electron microscope (SEM) Jeol JSM-7600F, and element determination was analyzed with energy dispersive X-ray (EDS) spectrometer. The 2D and 3D nano-profile of IDEs and MoSe_2_ were analyzed with NV-2000 Universal non-contact surface profiler. The Raman shift of MoSe_2_ nanoflakes are provided in Supplementary information. The 2D Nano-profile of IDEs is shown in Fig. 1d, which ensures that electrodes were correctly fabricated with screen-printer with surface roughness ~ 2.55 μm. The 3D Nano profile of IDEs are shown in Fig. 1e, representing the average height of IDEs ~ 9.68 µm. The surface morphology of MoSe_2_ nano-flakes was observed at 500 nm a shown in Fig. 2a. The EDS spot profile of MoSe_2_ nano-flakes is shown in Fig. 2b, which confirms the presence of Mo and Se peaks with atomic percentage 33.1% and 66.9%, respectively. The EDS mapped image of MoSe_2_ with magnification level of 500 nm as shown in Fig. 2c confirms the presence of Se L series as shown in Fig. 2d, and Mo L series in Fig. 2e. The 2D nano-profile of MoSe_2_ flaks is shown in Fig. 2f, which confirms the active layer roughness ~ 145.82 nm. The average height profile of MoSe_2_ flaks ~ 0.81 µm is shown in Fig. 2g.

### Conduction mechanism

**Figure 3:**
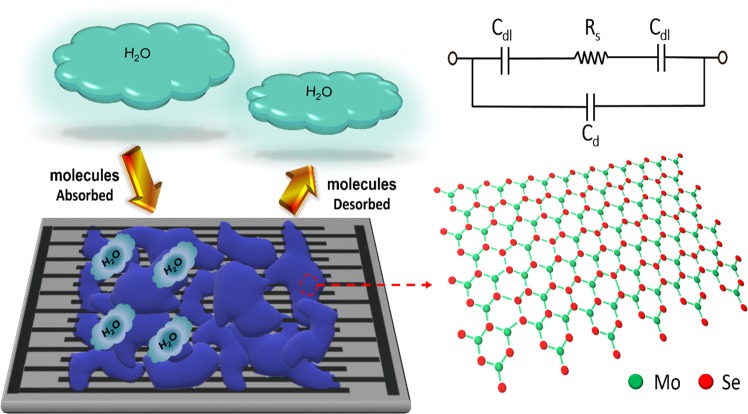
Sensing mechanism based on MoSe_2_ nano-flakes and its equivalent circuit.

The MoSe_2_ nano-flakes respond to humidity and dielectric constant of the film increases as compared to the dry film and ionic current flows through the sensor. With exposure of sensor to humidity and the hydroxyl ions, water molecules get absorbed into the thin film of MoSe_2_ and ionic conduction paths are formed between MoSe_2_ nano-flakes. This results in decrease of overall sheet resistance. Figure 3 shows random sized and randomly placed nano-flakes above the IDEs. Additionally, void spaces are created between the layers of MoSe_2_ nano-flakes, which not only increase the Molybdenum and Selenium edges for H and O bonds, but also water penetration between the layered formation becomes easy as compared to Bulk and Nano-flower solid centre cores^12^. Absorption of water molecules shifts the Fermi energy from semiconductor nature of MoSe_2_^26,43^ towards conduction band^12^. Increasing the number of free electrons and hence a higher electrical conductivity of the sensing sheet^12,44^. Enhanced surface roughness of MoSe_2_ nano-flakes helps to increase the sensitivity of the sensor, as earlier reported for same family of materials^13^, making a large surface area resulting in higher molecular bonding rate. Further explanation of sensing mechanism is added in the supplementary information. In other words, with the increase of humidity level the capacitance of the device increases as a result the impedance of device decreases.

The equivalent circuit^45^ given in Fig. 3, *C*_*dl*_ represents the double layer capacitance, *R*_*s*_ shows the sheet resistance, and *C*_*d*_ is the dielectric behavior of the humid air above sensor surface. The sheet resistance and capacitance are calculated as Eqs. (1) and (2)^45^.

1 $${R}_{s}=\frac{1}{nlk}\frac{2K\left(sin\left(\frac{\pi {w}_{sp}}{2L}\right)\right)}{Kcos\left(\left(\frac{\pi {w}_{sp}}{2L}\right)\right)}$$

2 $${C}_{dl}=nl\varepsilon \frac{2K\left(sin\left(\frac{\pi {w}_{sp}}{2L}\right)\right)}{Kcos\left(\left(\frac{\pi {w}_{sp}}{2L}\right)\right)}$$

Here, *n* is the number, *l* is the length of IDE fingers, *k* is the sheet conductivity, *w*_*sp*_ is the electrode spacing, *L* is the characteristic length equal to electrode spacing +width, and *ε* is the relative permittivity. The resistance and capacitance thus become a function of the electrode spacing ratio *w*_*sp/*_*L*. At low frequencies the impedance behavior is dominated by capacitive effect especially due to *C*_*dl*_. Between frequencies 1–10 kHz the impedance is a combination of resistive as well as capacitive effect^45,46^. Therefore, calculations were performed at 1 kHz to incorporate the change in capacitive behavior under variating humidity conditions.

### Measurement setup

**Figure 4:**
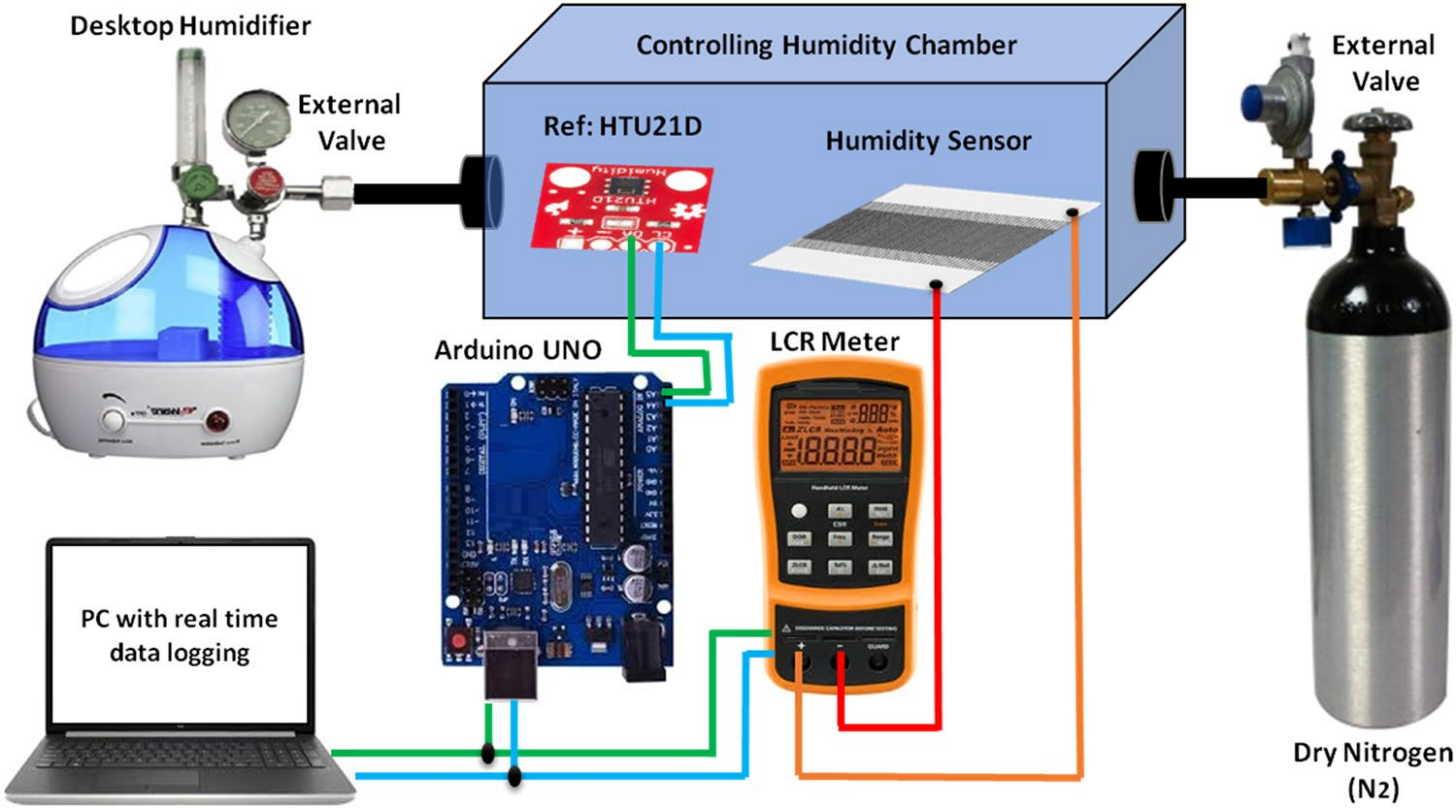
Experimental setup block diagram.

To keep experimental setup, an airtight homemade box was used as humidity test box. To reduce the error margin a commercial HTU21D sensor with resolution of 0.04% RH with accuracy of ±2% RH with time response of <5 s and temperature coefficient of −0.15% RH/°C was used as reference sensor. The measurement setup included a KEYSIGHT Digital U1700C hand held LCR meter with an Arduino UNO as control setup, and for increase and decrease in humidification, humidifier and dry nitrogen (N_2_) were utilized respectively as shown in Fig. 4. For data acquisition, personal computer (PC) was used while the fabricated sensor data was analyzed on built-in-software of KEYSIGHT Digital U1700C hand held LCR meter. The reference logging data were performed on cool term software with OriginPro 8.0 for plotting graphs. Both the Arduino UNO and LCR meter were connected through Universal Serial Bus (USB) Port with PC for automatic data logging. Commercial humidifier increases the humidity level from 0% to 90% inside the humidity box while dry N_2_ with external control valve dehumidifies the box. Experiments were performed from 20 to 30 °C with a step size of 5 °C. For transient response measurement, a sudden increase from 0 to 90% RH and sudden decrease from 90% to 0% RH was executed. Figure 4 represents the complete block diagram of experimental setup.

### Simulation setup

The sensor based on three-dimensional structure was designed on COMSOL Multiphysics 5.3a software platform. Two physics modules/disciplines were assigned to the model namely electrostatics and moisture transport. Impedance calculations were performed through electrostatics module while the moisture absorption, desorption, and change in relative permittivity of sensing layer are studied through the moisture transport module. Materials were assigned to individual layers, while the parameters for sensing layer were defined through material property functions. To minimize simulation time, a free-tetrahedral meshing topology was utilized with largest possible sizing for relatively reduced number of degrees of freedom. A time dependent study analysis was chosen for the investigation of relative humidity (RH) for better understanding of absorption process. The open surface area was exposed to different humidity levels ranging from 0 ~ 90% RH with a step size of 5%. As the humidity level increases the vapor concentration (mols/m^3^) increases, hence absorbed water content by the sensor does not remain at equilibrium and water molecules start to diffuse in the sensing layer. This phenomenon is shown in Fig. S7a,b of supplementary information, where water content at 0% RH and 90% RH levels is represented, respectively. Simulation and design details are provided in supporting information file.

## Results and Discussion

**Figure 5:**
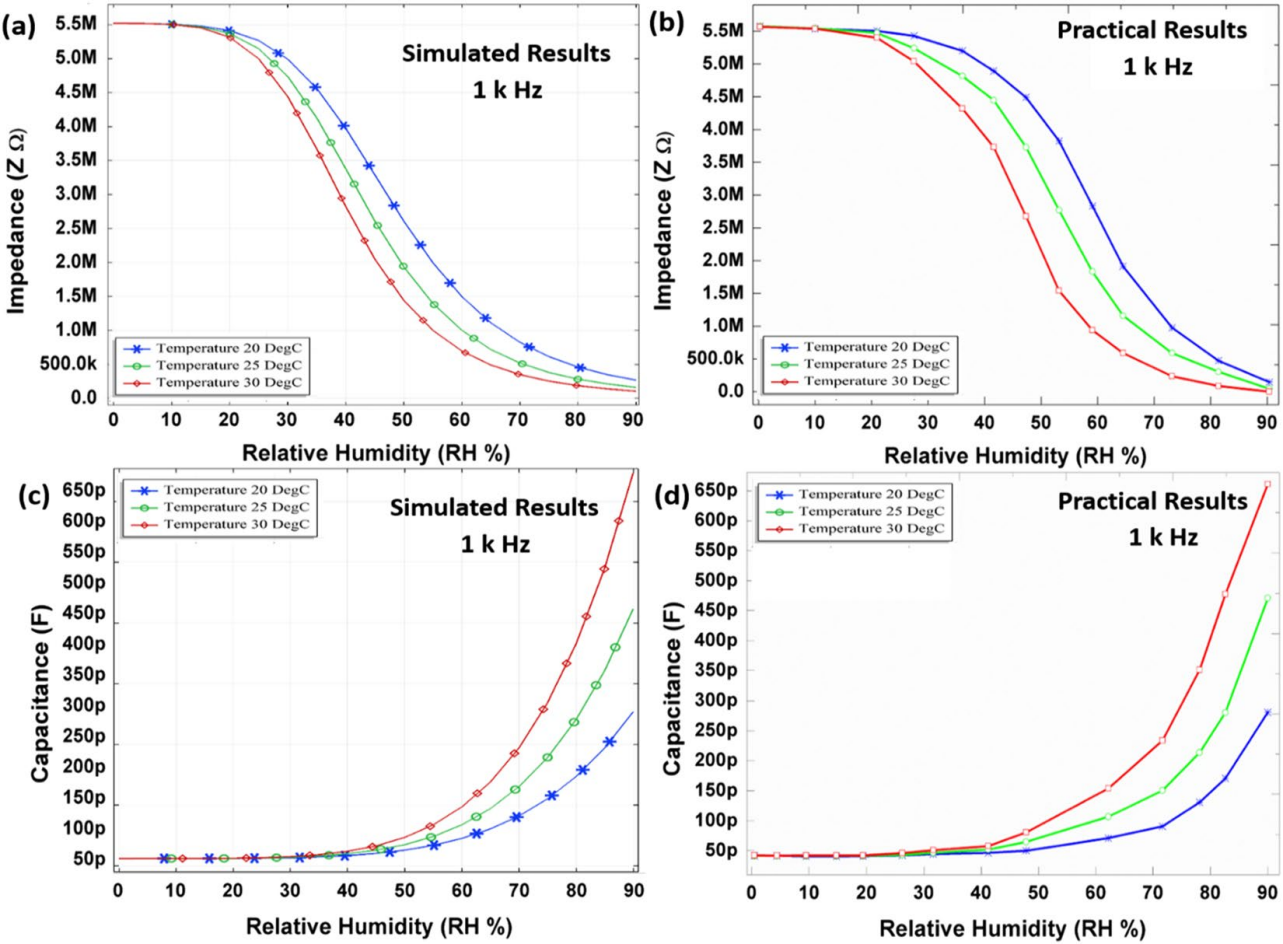
(**a**) Simulated impedance analysis and (**b**) practical impedance analysis of MoSe_2_. (**c**) Simulated capacitance and (**d**) practical capacitance analysis of MoSe_2_.

The effect of absorption of H_2_O molecules on electrical properties of MoSe_2_ sensing layer was studied by impedance and capacitance measurements in a homemade airtight box utilized as humidity chamber. In this experiment, a step wise change in temperature (*T*) and RH were performed in a controlled way (*T* range 20–30 °C with 5 °C step size and RH range of 0–90%). Multiple sensors were fabricated and their response was recorded as shown in Fig. S10 of Supplmentary Information. which shows the sensor can be mass produced and is highly reproducable. Four types of response analysis included impedance, capacitance, hysteresis and transient response discussed in subsections below:

Impedance response. As the temperatures rises, the kinetic energy of molecules increases as well as gas pressure, and *vice versa*, the diffusion rate of water molecules in MoSe_2_ changes with the variating ambient temperature. The gas pressure is expressed as in Eq. (3):

3 $$P{V}_{vol}=nRT$$

where, *P* is the vapour pressure, *T* is temperature, *n* is number of moles, *V*_*vol*_ is volume, and *R* is ideal gas constant. Deriving from Eq. (3), the average kinetic energy (*KE*_*avg*_) dependence on temperature can be calculated as in Eq. (4):

4 $$K{E}_{avg}=\left[\frac{1}{2}m{v}^{2}\,\right]=\frac{3}{2}kT$$

where, *m* is the mass, *v* is the velocity of molecules, and *k* is a ratio of gas constant over Avogadro’s number. The above equations are related to translational energy of molecules. As the kinetic energy of gas molecules increases with increase in temperature, even at lower RH the water molecules diffuse into the senstive layer and cause reduction in impedance. To analyze this dependence, different temperature levels ranging from 20 to 30 °C were simulated with a step size of 5 °C, which explain higher intermolecular interactions and binding with MoSe_2_ layer due to higher kinetic energy of water molecules. The impedance based simulated and practical results are presented in Fig. 5a,b. The volcano plots suggest^47,48^ a higher exchange current for MoSe_2_ for the above mentioned phenomenon. Thus, increase in humidity decreases the impedance of the sensing layer. MoSe_2_ general impedance is given in the Eq. (5).

5 $$Z=R+\frac{1}{j2\pi fC}$$

where, $j=\sqrt{-1}$, *f* is the frequency, *R* is the resistance of wires, and *C* is the capcitance. In this study, the measured and theoretical impedance are described as absolute value ($|{\rm{Z}}|$) without a phase information from Eq. (5). The impedance changes slightly till 20% RH due to low concentration of H_2_O molecules, and lesser number of molecular interacting with sensing layer. Impedance rapidly decreases as RH increases between 20% and 90%, which can be contributed to adequate number of molecular contentration of H_2_O for interaction with the MoSe_2_ with enough free electrons contribuing to lower electrical resistance.

The high density of exfoliated nano-flake edges allows a semiconducting to metallic phase transition^31^. Exfoliated nano-flakes are more disorderly placed as compared to nano-flower structures^31^. Hence, a simple exfoliation process enhances the chemical reactivity of MoSe_2_ as compared to grown nano-structures with solid center cores^31^. The bond lengths of Se–H bond were calculated as 1.48 Å^30^ and the Se egde shows a very weak hydrogen binding energy^30^. MoSe_2_ has a lower *φ* and its Fermi energy lies closer to normal hydrogen electrode^30^. This allows a much easier exchange of outer most electron at the Se edge. Above 90% RH, the proposed sensor reaches saturation of intermolecular interactions. The details for our simulation are described in the Supplementary information file. The small difference between the simulated and practical results was observed, hence we can say that the theoretical values were matched quiet well with the practical ones in aspect of design. It is attributed to large surface area, void spaces and surface roughness of MoSe_2_ nanoflakes, which contributes to the higher molecular bondings, while larger surface area gives a higher saturation limit in RH as earlier reported for same family materials^13^.

### Capacitance response

**Figure 6:**
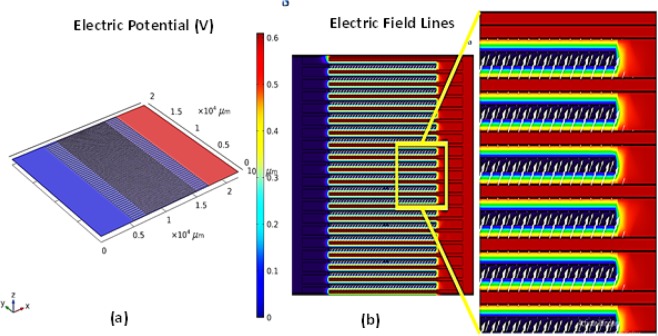
(**a**) 0.6 V electric potential applied across IDEs. (**b**) Electric field between IDEs.

The sensing layer is a semiconductor material, and its dielectric constant changes with the amount of water content absorbed by the layer. This change in dielectric constant, here, was measured as a change in capacitance by energizing the electrode structure. The capacitance based theroetical and practical results are presented in Fig. 5c,d, repectively, at 1 kHz with temperature levels ranging from 20 to 30 °C with a step size of 5 °C. The capacitance of humidity sensor increases while impedance decreases. Therefore, an electric potential of 0.6 V was applied across the IDEs to energize the sensor structure, as a result leakage current flows through IDEs and the resistance of the capacitor decreases. The effective capacitance (*C*_*eff*_) of the humidity sensor is presented in Eq. (6).

6 $${C}_{eff}={\varepsilon }^{\ast }{C}_{o}=({\varepsilon }_{r}-j(\gamma /2\pi f{\varepsilon }_{o})){C}_{o}$$

where, ε^*^, *ε*_*r*_, *ε*_*o*,_ *C*_0_, and γ are the complex dielectric constant, relative permittivity of ideal capacitor, dielectric constant of free space, expected capacitance, and conductance, respectively as given in Eq. (6). Water molecules make bonds on the Mo and Se edges of the nano-flakes and this causes change in relative permittivity of the sensing layer with higher hydrogen bonds created under higher water concentration. Figure 6a represents the simulation results of the applied electric potential across the IDEs, and an electric field, consequently, is formed between the electrodes as represented in Fig. 6b. The increase in dielectric constant of the MoSe_2_ layer, due to increase in water concentration, initiates a unique phenomenon that more charge is stored at electrode surfaces. This increased capacity of storing charge enhances the overall capacitance and represented by Eqs. (7) and (8), where, *Q* is the terminal charge, *V*_*bias*_ is the biasing or applied voltage, and *C*_*s*_ is the capacitance of the system. Further capacitance and energy related simuation results are discussed in the Supplementary information file.

7 $$Q={C}_{s}{V}_{bias}$$

8 $${C}_{s}=Q/{V}_{bias}={\varepsilon }_{o}{\varepsilon }_{r}A/d$$

Sensitivity and sizing tradeoff. Sensitivity of the sensor here is defined by the Eqs. (9) and (10)^49–52^. Here, *Z*_*u*_, *Z*_*l*_*, C*_*u*_, and *C*_*l*_ are the upper and lower limits of the magnitude of impedance and capacitance, respectively, and *RH*_*u*_ and *RH*_*l*_ are the upper and lower limits of relative humidity, respectively. These Eqs. (9) and (10) are employed to calculate the sensitivities of the sensor.

9 $$S=\frac{({C}_{u}-{C}_{l})}{(R{H}_{u}-R{H}_{l})}\times 100$$

10 $$S=\frac{({Z}_{u}-{Z}_{l})}{(R{H}_{u}-R{H}_{l})}\times 100$$

**Figure 7:**
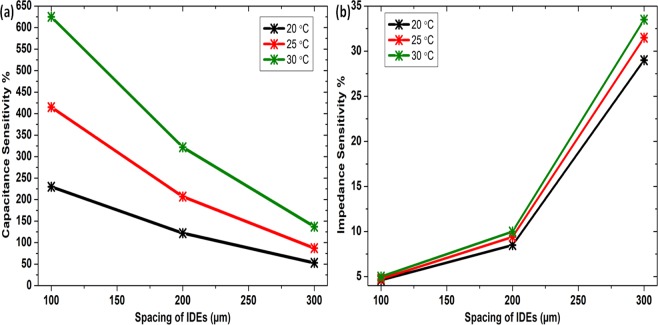
Sensitivity w.r.t. IDEs spacing of (**a**) capacitive and (**b**) impedance.

Figure 7a shows sensitivities calculated for capacitance measurements and Fig. 7b shows sensitivities for impedance measurements for temperature range from 20–30 °C obtained at different IDEs spacing in the range 100–300 µm. A tradeoff is observed from sensitivity calculations, if tried to further increase the capacitance response the sensitivity of the sensor in terms of impedance reduces and *vice versa*. It directly follows the below mentioned behavior in Eq. (11), capacitance, *C* is inversely proportional to the impedance, *Z*. Hence, a spacing size of 100 µm is chosen having highest capacitance in pF range and lowest impedance in MΩ range.

11 $$Z\propto 1/C$$

**Figure 8:**
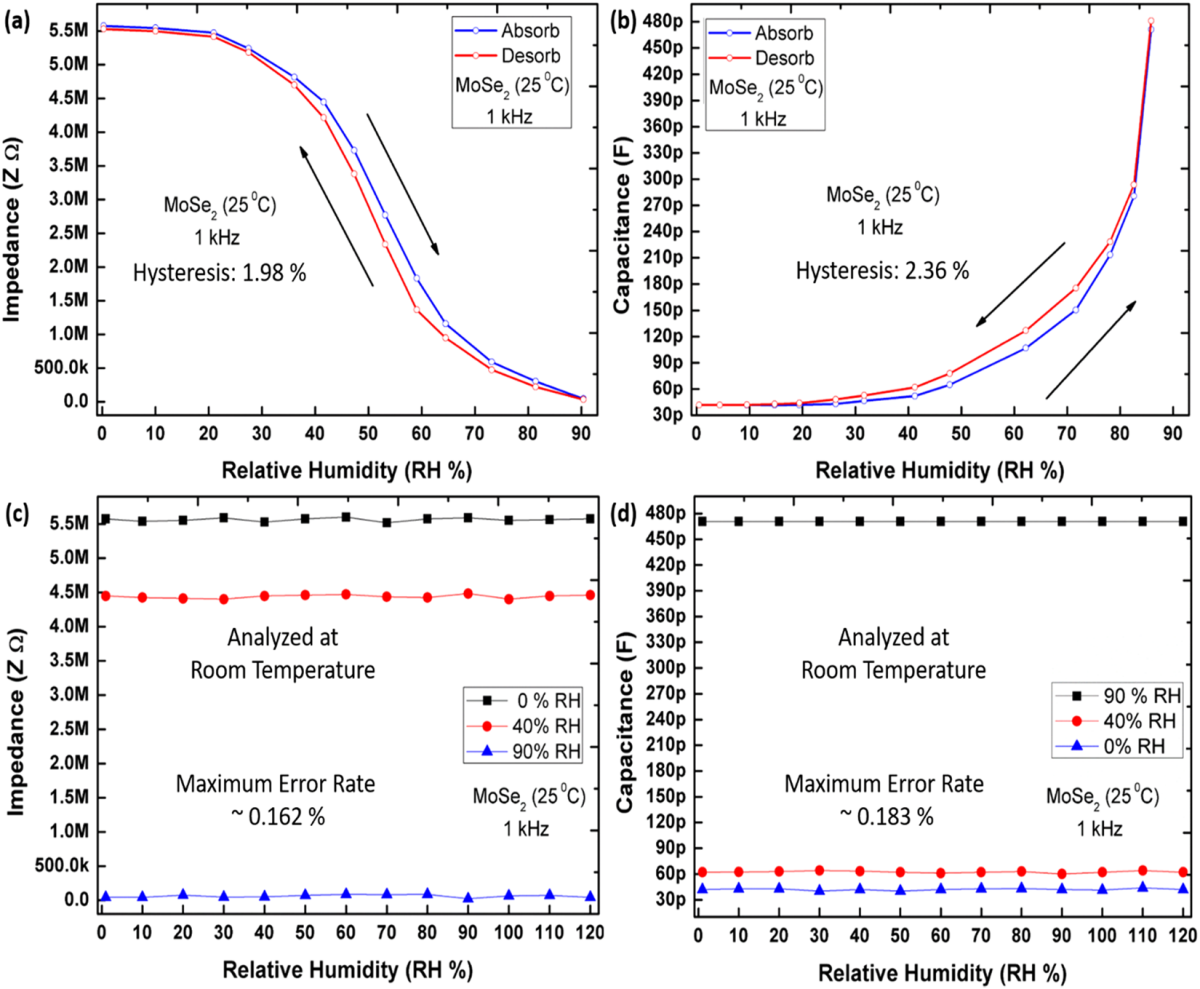
Hysteresis and Stability (**a**) Impedance hysteresis study, (**b**) capacitance hysteresis study, (**c**) impedance stability, and (**d**) capacitance stability.

Hysteresis and stability analysis. Hysteresis is caused due to trapping of charges between the molecular gaps, which is injected from/to the interfaces between semiconductor, substrate, and adsorbates. Hysteresis effect in MoS_2_ has been reported due to major S–H atom interatctions and water molecules are considered to be the major cause of hysteresis. Water molecules form a large electric dipole of ~ 1.8D and can align under electric field to form polar molecular structure. This structural alignment causes different charge trap densities and hence causing hyesteresis^20^. Similar is the case for MoSe_2_ which has much weaker Se–H bond. The impedance and capacitance based hysteresis characteristics are shown in Fig. 8a,b, respectively. Initially, the sensor was placed at 0% RH, then humidity level was ramped from 0 to 90% RH, and back from 90 to 0% RH. Both impedance and capacitance of the sensor were recorded during adsorption and desorption cycles. Average hysteresis was calculated by using Eq. (12).

12 $$Average\,Hysteresis=[\mathop{\sum }\limits_{k=0}^{n}({y}_{k+1}-{y}_{k})/({y}_{max}-{y}_{{\rm{\min }}})]/n$$

Here, ${y}_{k}\,k=\{0,\,1,\,\cdots ,\,n\}$ is impedance at *k*th test point, and *y*_*max*_ and *y*_*min*_are maximum and minimum impedance values, respectively, in number *n* test points data. The percentage hysteresis during humidification and dehumidification of impedance and capacitance are stated on each graph curvature presented in Fig. 8a,b, respectively. Impedance and capacitance values during desorption cycle lower RH (0 ~ 30%) show no hyeteresis due to very low H_2_O concentration. While, the upper (80 ~ 90%) values are overlapping which again shows no hysteresis, due to saturation of H_2_O in atmosphere. In range of 30–80% RH the curve follows different impedance and capacitance paths contributed to varying H_2_O concentration, and hence different Se–H interactions. The impedance and capacitance stability and repeatability was investigated by keeping the humidity sensor in different ambient conditions of 90% RH, 40% RH (open air response) and 0% RH for 120 min as shown in Fig. 8c,d. The stable impedance and capcitance response was recorded with maximum error rate of ~ 0.162% and 0.183%, respectively.

**Figure 9:**
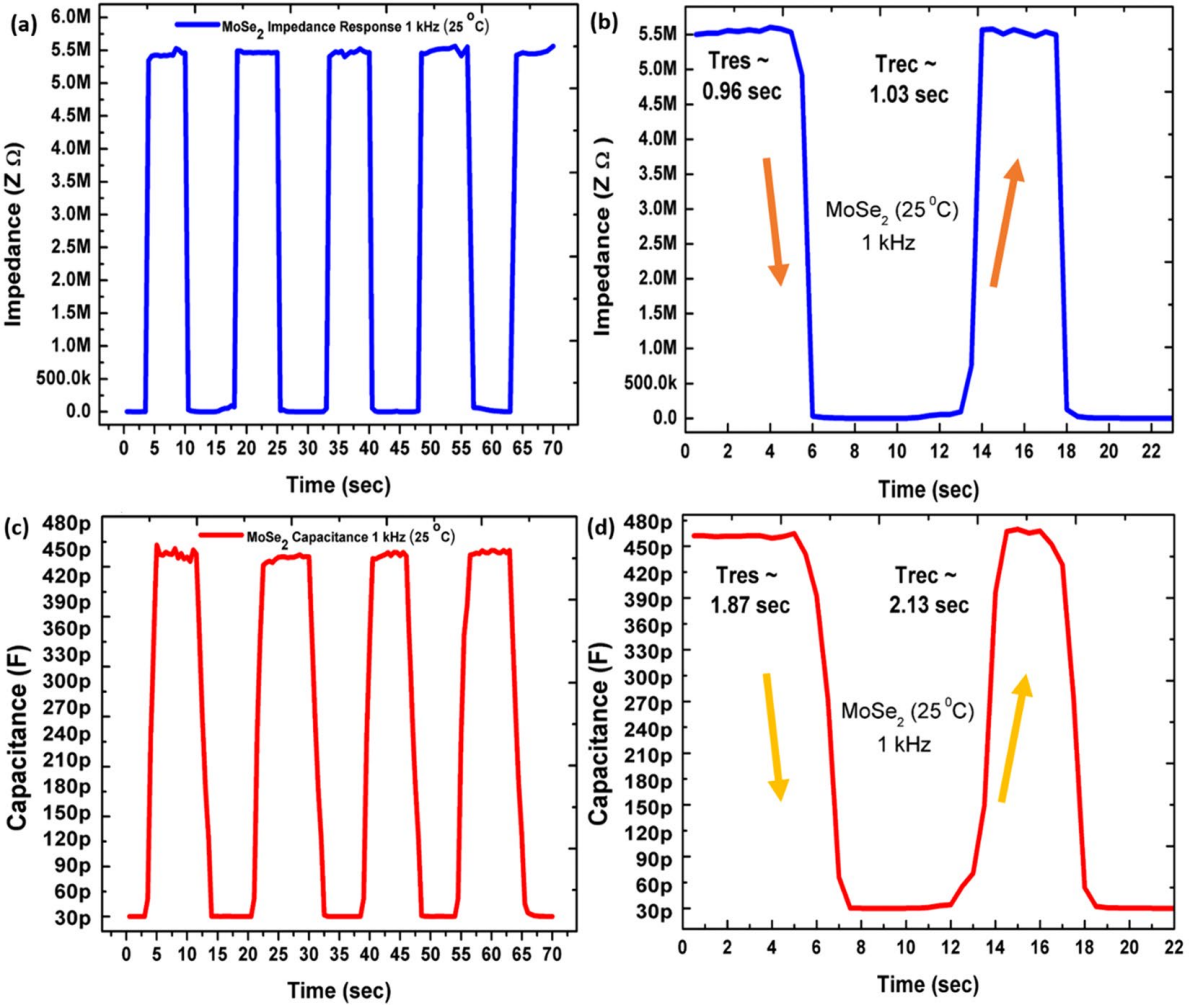
(**a**) Impedance response on longer scale showing, (**b**) reponse time of 0.96 s and recovery time of 1.03 s. (**c**) Capacitance response on longer scale showing, (**d**) response time of 1.87 s, and recovery time of 2.13 s.

**Figure 10:**
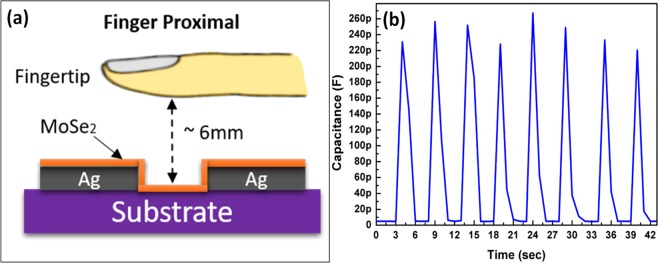
Contactless switch application (**a**) Experimental setup for contactless sensing of a proximal human fingertip. (**b**) MoSe_2_ sensor time-resolved proximal human fingertip test under ambient air conditions.

Transient response. As shown in Fig. 9, the recorded transient response of the sensors facilitates investigation of response time (*T*_*res*_) and recovery time (*T*_*rec*_) of the sensor during humidification and dehumidification. The transient response indicates sensitivity of sensor on sudden change in humidity levels in environment. As shown in Fig. 9a the impedance response of humidity sensor on longer scale showing *T*_*res*_ ~ 0.96 s and *T*_*rec*_ ~ 1.03 s respectively, as shown in Fig. 9b. The capacitance response of humidity sensor on longer scale As shown in Fig. 9c showing *T*_*res*_ ~ 1.87 s and *T*_*rec*_ ~ 2.13 s, respectively, as shown in Fig. 9d. To compare the findings of MoSe_2_ based humidity sensor with other reported sensors, Table 1 shows the comparison on the basis various characteristics include fabrication technique, response time, recovery time, range, materials, and the sensitivity of the sensors. The graphene and ZnO composite sensor^53^ is targeted as a wide range from 0 to 85% RH, but presented a mismatch in response and recovery times. In Table 1, the bio-compatible research works^54,55^ have the advantage of wide application range, but they have a large difference in response and recovery times of approximately 11 and 4 times, respectively. A big difference in response time requires a complex control structure and restrict its usage for real time applications. The fourth listed work is a tough competitor with respect to response and recovery time; however, it has a range from 15% to 78%^39^. The best possible real time monitoring sensor for health services and applications (breath test)^11^ has a narrower range from 30% to 80% RH. Sixth in the list presents chemical etching and dispersion process that requires extra care while handling with acids^56^, and cannot be directly printed. Seven, eight and nine listed works^13,57,58^ belong to the same material family of MoSe_2_, but present a far inferior response to MoSe_2_ in term of range and in response and recovery time. Tenth, inkjet-printed black phosphorus has a wide range, but a slow response time of 4.7 s^59^. Lastly, MoSe_2_ nano-flower structure was fabricated with a wide range of 0–97% RH, but again lacked in response and recovery times^12^. Here, we have targeted a wider range between 20% and 90% RH with fast and equivalent time response of 1 s approximately, which makes it suitable for real time monitoring and control applications. The difference between response and recovery time is small, hence this designed sensor is suitable with a simpler control and monitoring circuitry. The presented sensor gives a maximum detection of 30 breaths/min, keeping in mind that average breathing rate at rest in adults is 12–18 breaths/min^60^. A human breath detection test is performed and video is provided in Supplementary information. The ultrasensitive moisture response of MoSe_2_ sensor allows its utility in applications like contactless switch as shown in Fig. 10a. The humidity sensor shows a rapid real-time change of capacitance (up to 260 pF) at 1 kHz after placing a finger approximately 6 mm above the device as shown in Fig. 10b. The ultrasensitive capacitive response of the MoSe_2_ humidity sensor allows the capture of moisture from surface of human fingertip. If the finger is covered with tape, the sensor shows no response to the presence of the finger.

**Table 1:** Comparison Table.

S. No	Article Reference	Sensor Type	Fabrication Process	Range	Response Time	Recovery Time	Material Type	Sensitivity
1	^53^	Impedance	Inkjet Printing	0–85%	1 s	2 s	Graphene and ZnO Composite	High Sensitivity > 60%
2	^54^	Impedance	Screen Printing	0–80%	1 s	10.75 s	Single Cell thick Onion membrane	High Sensitivity > 50%
3	^55^	Impedance and Capacitance based	Inkjet Printing	0–90%	1.99 s	8.76 s	Egg shell membrane	High Sensitivity between 40–70%
4	^39^	Resistance	Spin Coating	15–78%	1.78 s	0.45 s	Lead free Cs_2_BiAgBr_6_	High Sensitivity > 60%
5	^11^	Impedance	Screen Printing	30–80%	30 ms	30 ms	Graphene Oxide 15 nm layer	Linear Response between 35–75%
6	^56^	Transmission light Power	Chemical etching and dispersion	20–80%	0.066 s	2.395 s	MoS_2_ coated etched single mode fibre	0.94–1.06 mW Change in Power Consumption
7	^57^	Resistance	Stamping	0–100%	30–40 s	12–50 s	VS_2_	Highly Sensitive > 50%
8	^13^	Resistance	Drop Casting	0–60%	9 s	17 s	MoS_2_	Between 10–50%
9	^58^	Resistance	Thermal Evaporation	40–80%	13 s	17 s	WS_2_	Between 40–80%
10	^59^	Capacitance	Inkjet Printing	11–97%	4.7 s	3.0 s	Black Phosphorus	High Sensitivity > 50%
11	^12^	Resistance	Hydrothermal Growth	0–97%	53 s	13 s	MoSe_2_ nano-flower	−74.41%
12	This Work	Impedance and Capacitance Based	Screen Printing	20–90%	0.96 s	1.03 s	MoSe_2_	High Sensitivity > 20%

## Conclusion

In this work, we demonstrated a highly stable, wide range and superfast humidity sensor based on MoSe_2_ utilizing a thin and highly rough surface sensitive layer for fast response. From this fabricated sensor, the impedance and capacitance change are recorded in range from 0% RH to 90% RH with different temperature levels ranging from 20 °C to 30 °C. The impedance and capacitance hysteresis recorded was <1.98% and <2.36%, respectively. The impedance stability recorded was with maximum error rate of ~ 0.162% and capacitance stability analyzed was with maximum error rate ~ 0.183%. The designed sensor shows good performance with fast response and recovery times of *T*_*res*_ ~ 0.96 s and *T*_*rec*_ ~ 1.03 s, respectively. Integration through inkjet printing technology is easily achievable for this sensor to monitor environmental and health services.

## Supplementary information

Supplementary information 1.

Supplementary information 2.

Supplementary information 3.

Supplementary information 4.
