# Supplementary material for: Printable Highly Stable and Superfast Humidity Sensor Based on Two Dimensional Molybdenum Diselenide
Source: Sci Rep. 2020 Mar 26;10:5509. doi: 10.1038/s41598-020-62397-x (PMC7099085; doi:10.1038/s41598-020-62397-x)
Supplement: Supplementary file 4 — Supplementary information 4. [file 41598_2020_62397_MOESM4_ESM.docx]

**Supplementary Information: Printable Highly Stable and Superfast Humidity Sensor Based on Two Dimensional Molybdenum Diselenide**

**Muhammad Awais, Muhammad Umair Khan, Arshad Hassan,**

**Jinho Bae and Tahseen Elahi Chattha**

**Polyethylene terephthalate (PET) substrate configuration**

A rectangular layer with dimensions 22 mm × 20 mm with length and width, respectively, is built as shown in **Figure S1**. Here, a polyethylene terephthalate (PET) is assigned as a substrate material for the sensor fabrication. Height of the substrate material is kept at 10 µm.

*
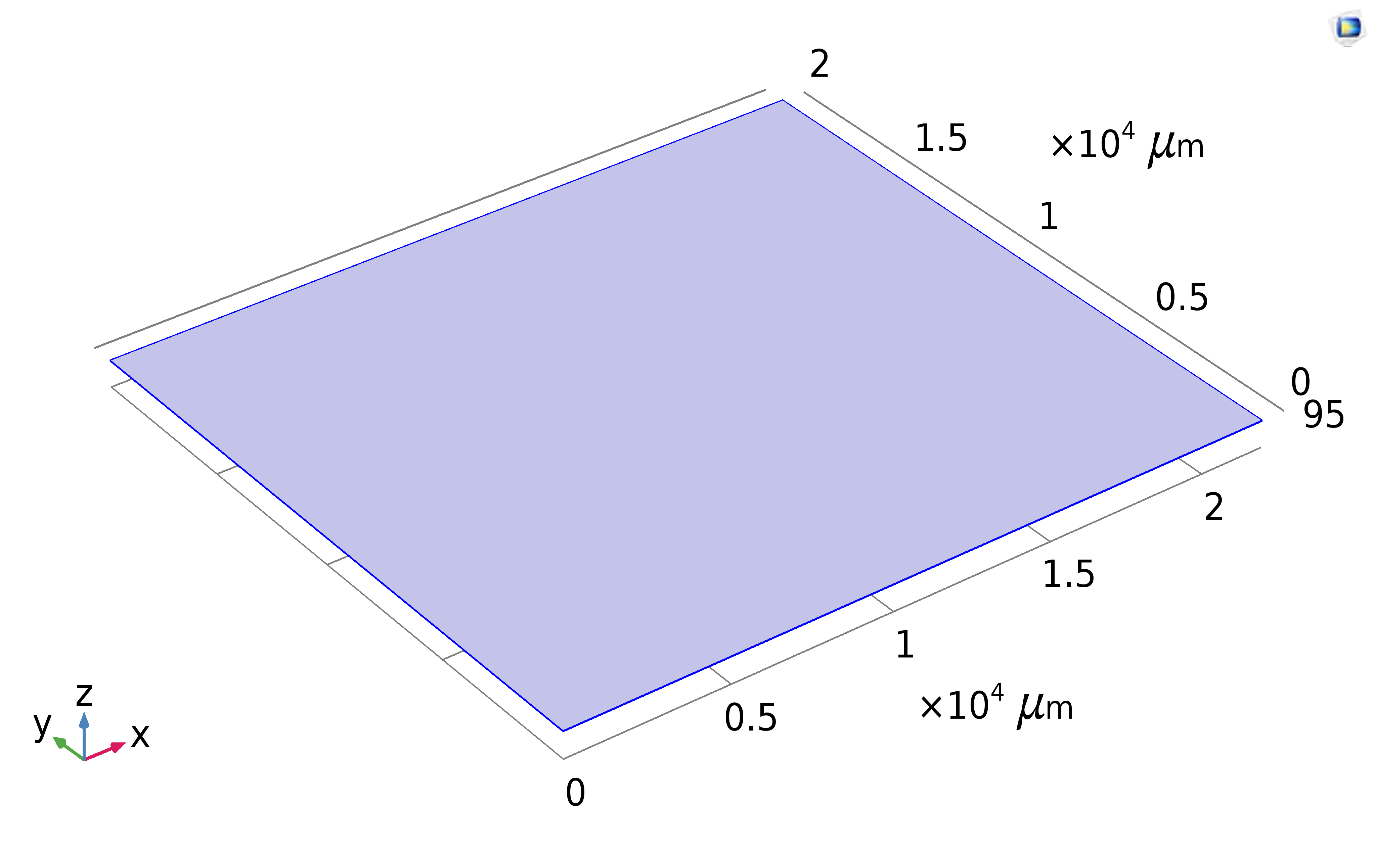
*

**Figure S1.** PET substrate formation.

**Silver (Ag) electrodes configuration**

Inter digitated electrodes (IDEs) are formed with rectangular plate size of 20 mm × 5 mm with length and width, respectively. The fingers of IDEs with length and width of 10 mm and 100 µm, respectively, are formed as highlighted in **Figure S2**. The height of the IDEs as kept at 10 µm with a spacing of 2 mm between the IDE ends. From materials section, silver as chosen and assigned to the IDEs.

**Figure S2.** Silver IDEs formation.

**Molybdenum diselenide (MoSe_2_) sensing layer configuration**

A sensing layer of MoSe_2_ with a height of 1.6 µm is uniformly formed on IDEs as highlighted (blue) in **Figure S3**. From materials section, MoSe_2_ is assigned as the sensing layer for humidity sensor.

**Figure S3.** MoSe_2_ layer formation.

**Moisture exposure**

To evaluate the effect of humidity, moisture in % for RH is exposed on the top of the sensor as highlighted blue color in **Figure S4**.


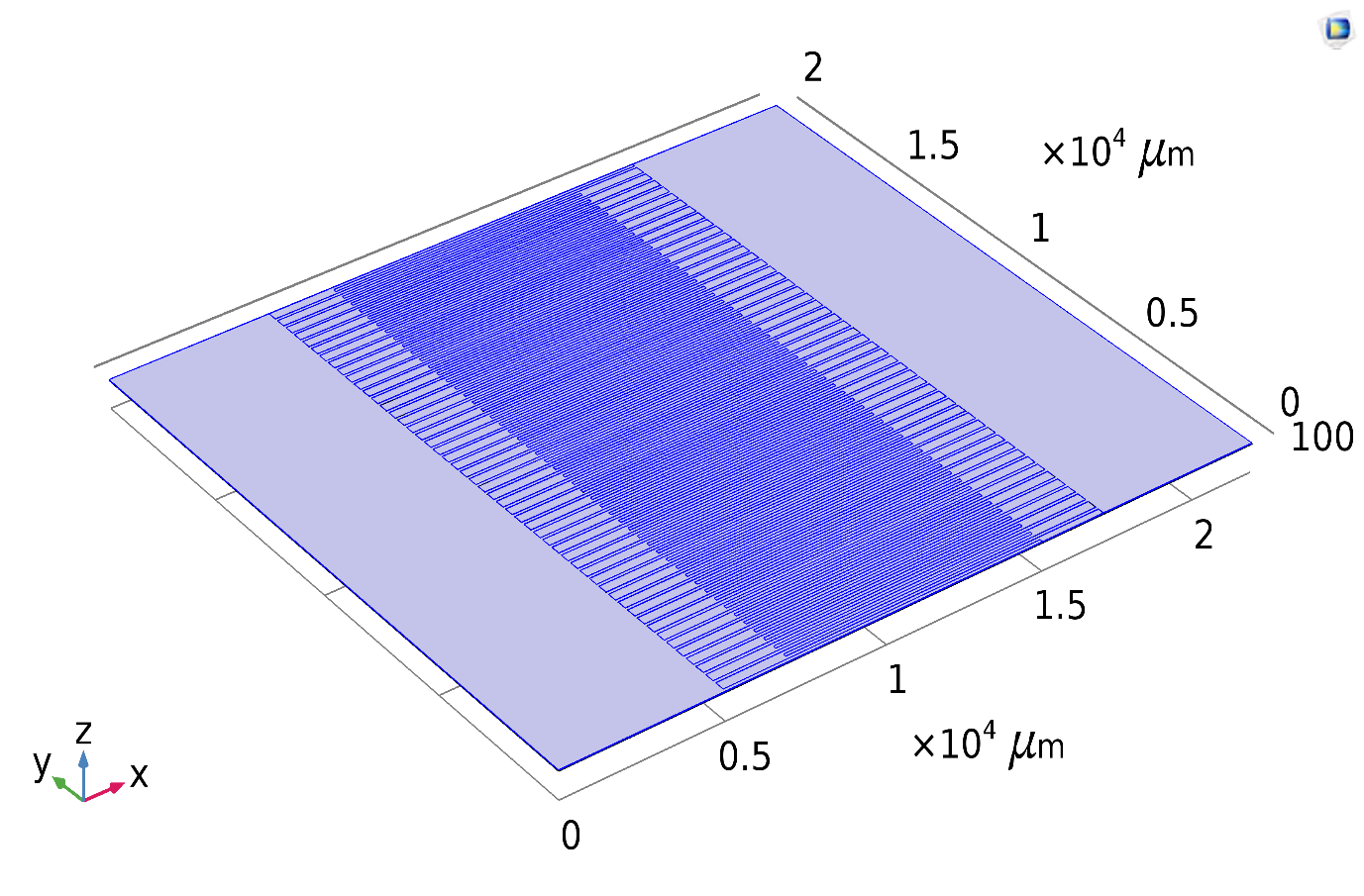
 **Figure S4.** Exposure to moisture in % RH.

**Mesh sizing and elements**

For size reduction and fast computation, free tetrahedron and triangular structures are utilized, and **Table S1**. represents the sizing and element information for meshing scheme.

**Table S1**. Mesh sizing and elements.

| **Description** | **Value** |
| --- | --- |
| Minimum Element Quality | 1.274E-9 |
| Average Element Quality | 0.02052 |
| Number of Tetrahedrons | 60567 |
| Number of Triangles | 34872 |
| Number of Edge Elements | 10137 |
| Number of Vertex Elements | 816 |
| Maximum Element Size | 11µm |
| Minimum Element Size | 1.54µm |
| Resolution at Narrow Edges | 0.1 |
| Maximum Element growth rate | 2 |

**Meshing implementation**

**Figure S5** represents the meshing topology formed after configuration of meshing scheme.


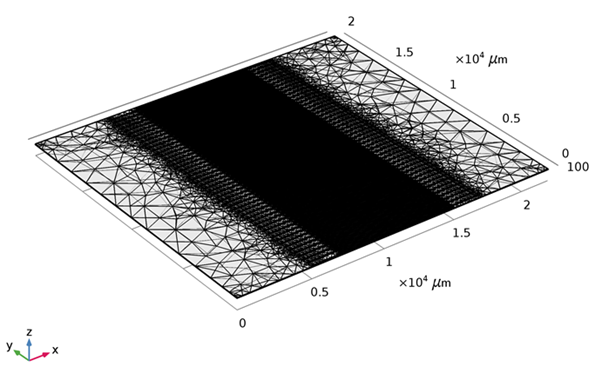


**Figure S5.** Mesh implementation.

**COMSOL theory and equations**

Generally, moist air is modeled as a mixture of two ideal gases. Dry air and vapor pressure are modeled by the ideal gas equations given below[^1^](#_ENREF_1):

$p_{d}=\rho_{d}R_{d}T$ (1)

$p_{v}=\rho_{v}R_{v}T$ (2)

where, $p_{d}$, $p_{v}$ are the partial pressures, *T* is the temperature, $\rho_{d}$, $\rho_{v}$ are the gas densities, and $R_{d}$, $R_{v}$ are the gas constants. Density in particular is calculated as

$\rho_{d}=\frac{m_{d}}{V}$ (3)

$\rho_{v}=\frac{m_{v}}{V}$ (4)

Here, $m_{d}$ and $m_{v}$ are masses of dry air and vapors, and *V* is the total volume that the gas mixture occupies. The specific gas constant can be calculated from the universal gas constant R = 8.314 J/molK and individual molecular weights as[^1^](#_ENREF_1)

$R_{d}=\frac{R}{M_{d}}$ (5)

$R_{v}=\frac{R}{M_{v}}$ (6)

Here, $M_{d}$ and $M_{v}$ are the molecular weights of individual gases in the mixture. Relative humidity is defined as the ratio of vapor pressure $p_{v}$ to the saturation vapor pressure $p_{v sat}$ and is given by[^1^](#_ENREF_1)^,^[^2^](#_ENREF_2):

$\varphi=\frac{p_{v}}{p_{v sat}}$ (7)

The saturation vapor pressure $p_{v sat}$, is a temperature dependent parameter and follows the expression as[^1^](#_ENREF_1)^,^[^2^](#_ENREF_2)

$p_{v sat}=614.3e^{\left\{ 17.06\left[ \frac{T-273.15}{T-40.25} \right] \right\}}$ (8)

COMSOL Multiphysics applies the relative humidity Eq. (7) directly and a sweep is created starting from 0–100% RH. Diffusion is generated and defined by the Fick’s law in COMSOL as[^3^](#_ENREF_3):

$J=\frac{D\partial c}{\partial_{x}}$ (9)

Here, *D* is the diffusion coefficient, and $\frac{\partial c}{\partial_{x}}$ is the change in concentration. Diffusion coefficient is in the range of 10^-5^ to 10^-6^ m^2^/s for gases[^4^](#_ENREF_4). Second physics applied is governed by simple electrical equations as[^5^](#_ENREF_5)

$\nabla.E=\frac{\rho}{\varepsilon_{o}}$ (10)

Where, $\rho$ is the charge density, and $\varepsilon_{o}$ is the permittivity of free space, and E is the electric field. The electric displacement field is given as[^5^](#_ENREF_5)

$D=\varepsilon_{o}E+P$ (11)

The Eq. (11) can be rewritten as Gauss’s law[^5^](#_ENREF_5)

$\nabla.D=\rho$ (12)

Therefore, for dielectric materials the most linear materials is given as[^5^](#_ENREF_5):

$D=\varepsilon_{o}\varepsilon_{r}E$ (13)

Here, $\varepsilon_{r}$ is the relative permittivity of the sensing layer dependent upon humidity.

**Ink Preparation and Spin Coating**


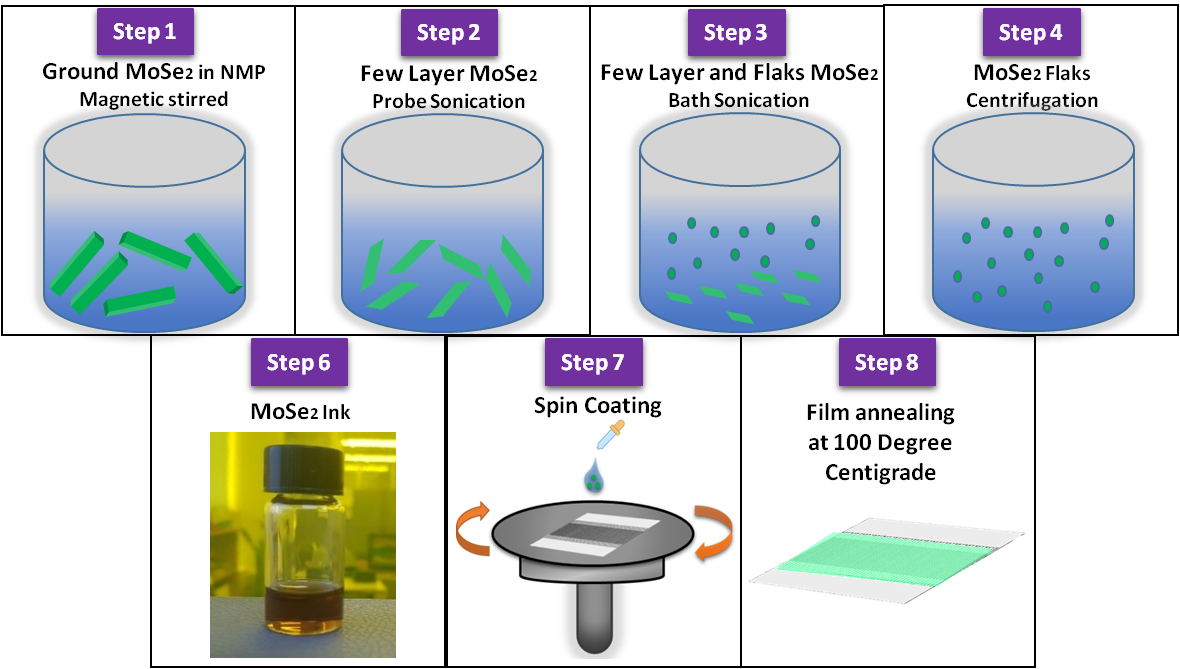


**Figure S6.** Wet grinding and spin coating process.

**Water content penetration in sensing layer at different humidity levels**

A 3D view of two simulated results are presented in the **Figure S7.** At 10% RH the figure shows very low concentration of water absorbed by the sensing layer represented in **Figure S7(a)**. **Figure S7(b)** shows a higher concentration at 90% RH in the centre where the MoSe_2_ layer is deposited.


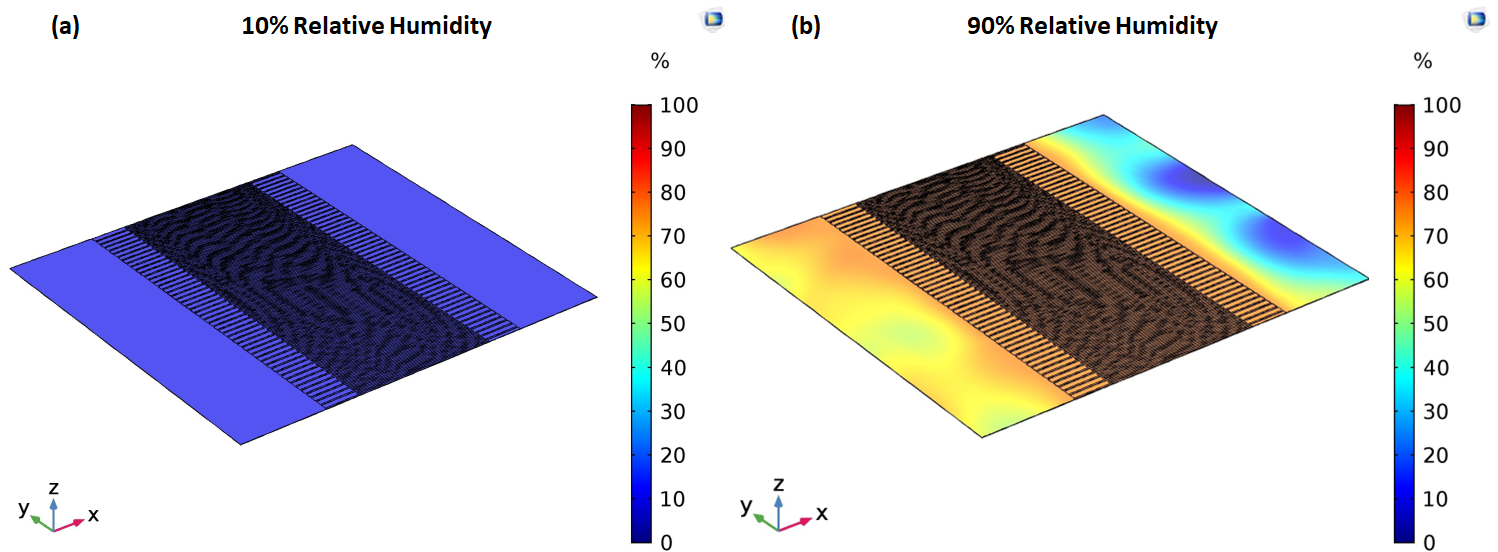


**Figure S7.** 3D view (a) Water content at 0% RH. (b) Water Content at 90% RH.

**Cross-sectional view of water penetration**

Water content penetration in sensing layer at different humidity levels: A patch of cross-sectional view of penetration of water at 0%, 75%, and 90% humidity levels are presented a, b, and c in **Figure S8**, respectively. In **Figure S8(a)**, as the air doesn’t contain any water content so the upper surface of the sensor represents water concentration of approximately 0%. In **Figure S8(b)** at 75% RH, the water molecules get absorbed and as MoSe_2_ layer has higher absorption rate so the higher water percentage is observed in the MoSe_2_ layer as compared to the surrounding layers. In **Figure S8(c)**, the water concentration reaches saturation and can be seen by a dark red color corresponding to 90% concentration in MoSe_2_ layer. The color-coding represents the molecular concentration in the sensor layers. The animation of penetration of water content over all humidity levels (% RH) is given in “Supplementary Video_Humidity Animation.mp4” as the Supplementary image.

**Figure S8.** Cross-sectional view of water penetration (a) 0% RH, (b) 75% RH, and (c) 90% RH.

**Electric energy**

Total electric energy consumed by the sensor structure with respect to increase in humidity level is represented in **Figure S9**. Here, this result explains as humidity increases more work is required to store more charges on electrode surface. The energy (*W*) is given by following [^6^](#_ENREF_6) equations as

$W=\frac{Q^{2}}{2C}$ (14)

Where, *C* is the capacitance and *Q* is the charge at electrode surfaces. Capacitance is also calculated by the stored charge on the electrode plates as given by

$C=\frac{Q}{\Delta V}$ (15)

Where, *∆V* is the difference of potential between the electrodes. From above equations, we can compute a direct capacitance and energy related relation and is given below as[^6^](#_ENREF_6):

$C=\frac{Q^{2}}{2W}=\frac{C^{2}\Delta V^{2}}{2W}$ (16)

Therefore,

$C=\frac{2W}{\Delta V^{2}}$ (17)

Capacitance increases, while the terminal voltage remains constant, the total electric energy in joules also increases. Animation of energy density is given in Supplementary image (“Supplementary Video_Energy Density.mp4”).


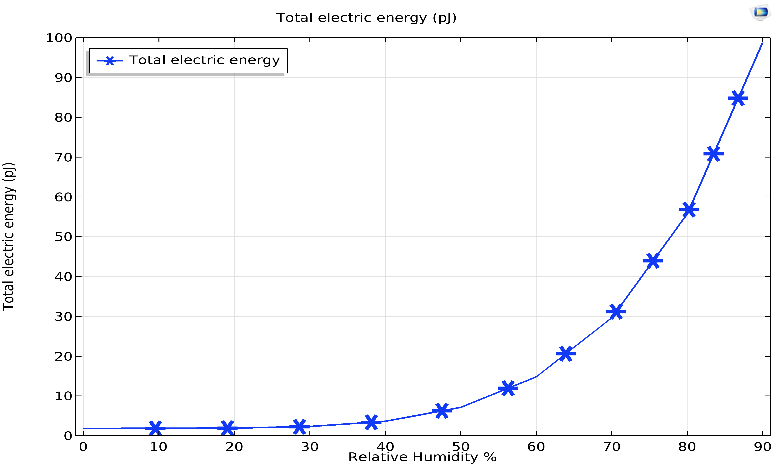


**Figure S9.** Electric Energy w.r.t %RH.

**Mass production of humidity sensors**

We fabricated multiple sensors to test repeatability and reproducibility of the sensor. For this purpose, capacitive response was measured of three different sensors. Temperature variation was added for these tests to assess variation in capacitive response. **Figure S10**. shows the response of the sensors. It can be seen that different sensors have well matched capacitive response and it can be reproduced for large scale production.


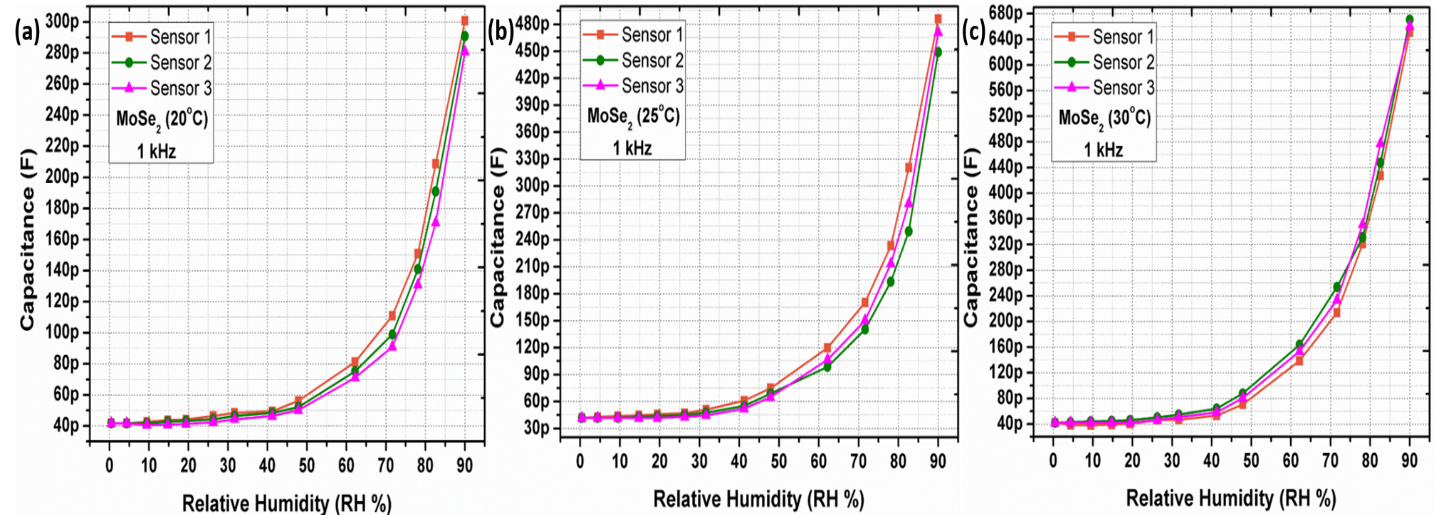


**Figure S10.** Temperature dependence capacitance response of Sensor 1, 2, and 3 on temperature range of (a) 20 ^o^C, (b) 25 ^o^C, and (c) 30 ^o^C.

**Sizing optimization**

Fixing the overall size of the sensor to 20 mm × 22 mm, we simulated three different models of the capacitive sensor with different IDE width and spacing. Width and spacing of 300 µm, 200 µm, and 100 µm are formed for Sensor 1, Sensor 2, and Sensor 3, respectively. All other design parameters are kept constants. The comb capacitance ($C_{comb}$) can be presented by the following equation[^7^](#_ENREF_7) as

$$C_{comb}=n\frac{\varepsilon tl}{d}$$

Here, *n* is the number of fingers created in one of the capacitors,$\varepsilon=\varepsilon_{0}\varepsilon_{r}$ is the permittivity, *t* is the thickness, *l* is the overlapping length of the electrode finger, and *d* is the gap between fingers.

As the gap between fingers is inversely proportional to the capacitance, clearly increasing the gap reduces the capacitance. The overall size of the sensors is kept constant, so change in IDE sizing changes the number of fingers of IDE. The *n* being directly proportional to capacitance has a direct relationship to the constituent capacitance. **Figures S11** and **S11** represent the capacitance calculated for Sensor 1 and Sensor 2, respectively, at temperatures between 20–30 ºC with a step size of 5 ºC.


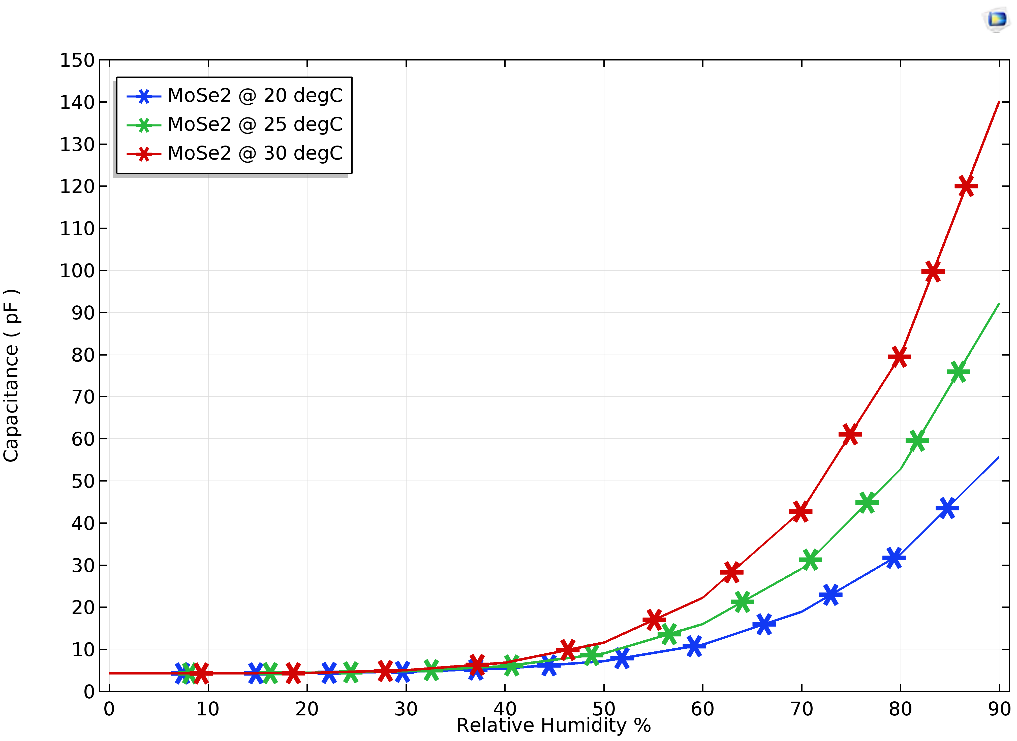


**Figure S11.** Sensor 1 with 300 µm spacing: capacitance at different temperatures.


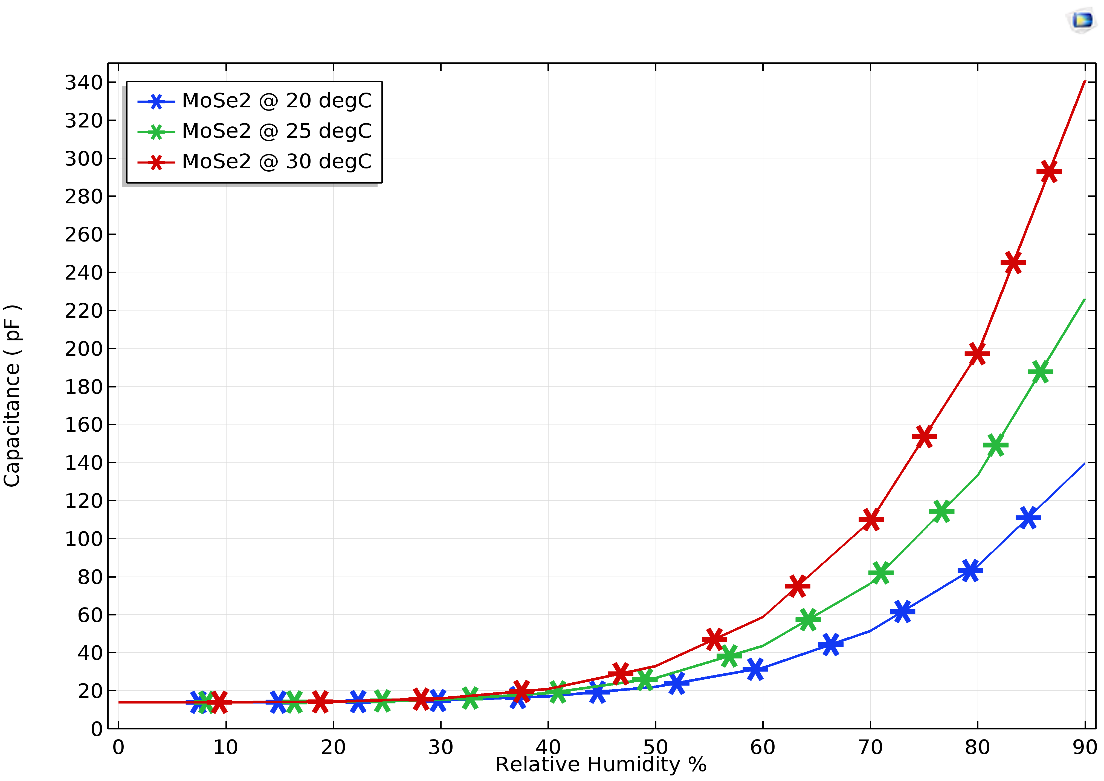


**Figure S12.** Sensor 2 with 200 µm spacing: capacitance at different temperatures.

Again, capacitance has an inverse relationship with the impedance gives higher impedance as represented in **Figures S13** and **S14**. Step size of 10% RH was taken to reduce number of steps for simulation between 20 ~ 30 ºC temperature range.


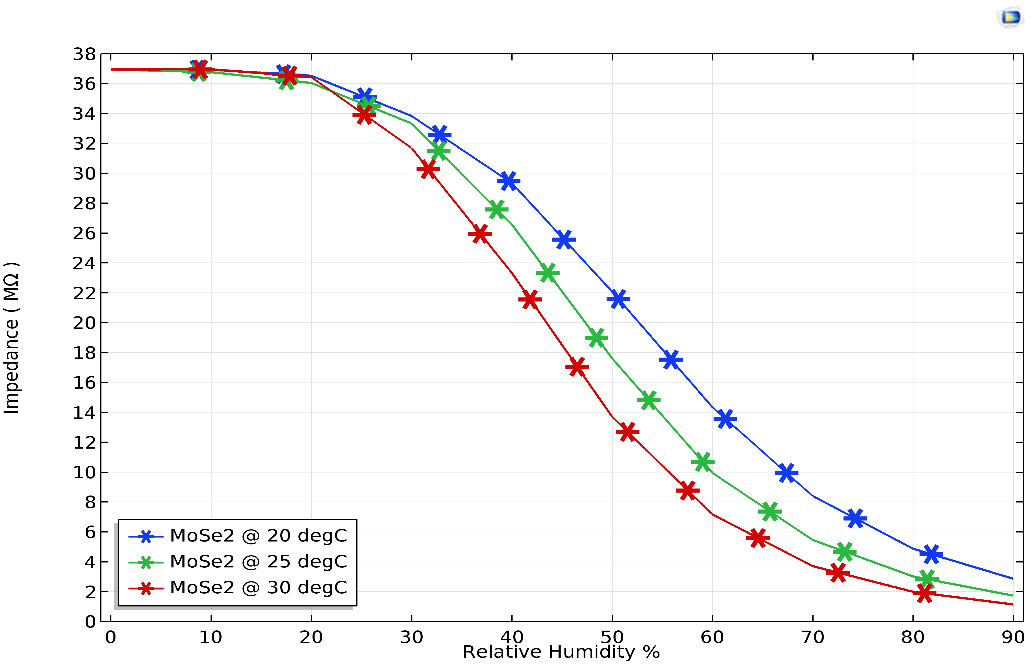


**Figure S13.** Impedance response of Sensor 1 (with 300 µm spacing).


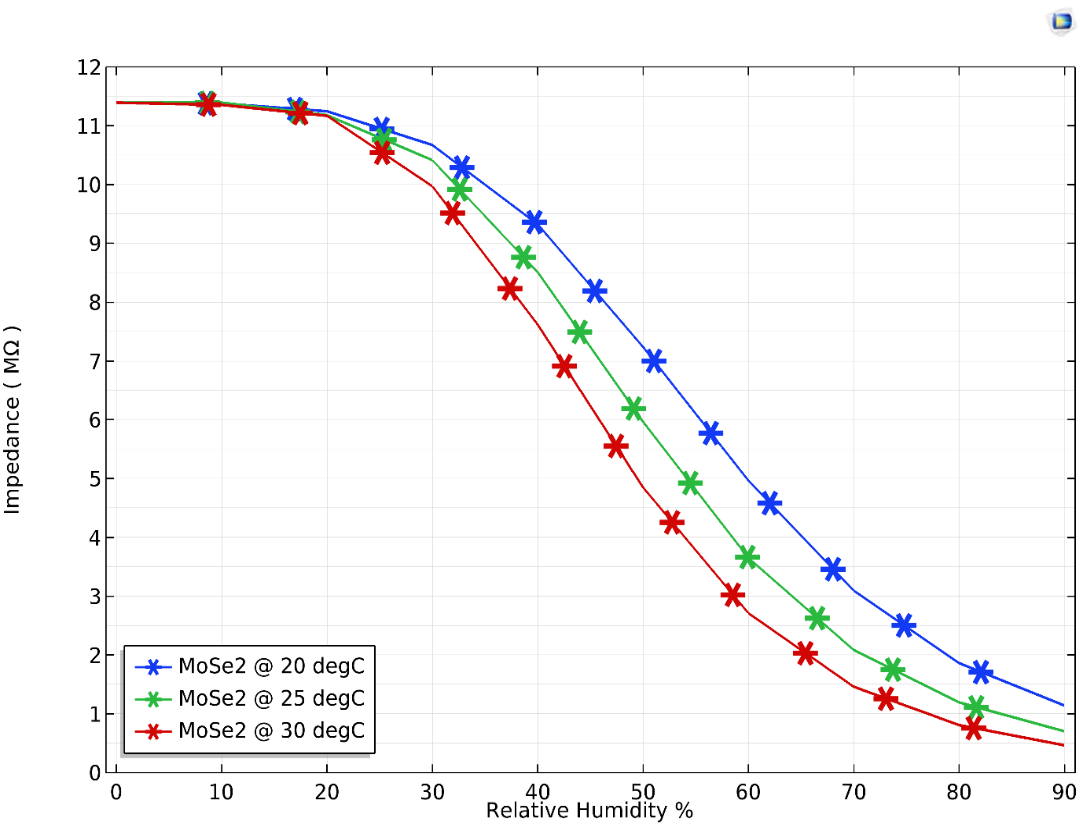


**Figure S14.** Impedance response of Sensor 2 (with 200 µm spacing).

Therefore, the above results suggest that reducing the spacing not only increases the base capacitance but also reduces the base impedance of the sensor. To remain in the ease of detection range, the highest capacitance and lowest impedance Sensor 3 is selected as discussed in the main work.

**Sensing mechanism**

The nano-flake sensing layer cross-sectional view is shown in **Figure S15**. Here, this view can give an insight, how the exfoliation process and nano-flake structure enhances hydrogen reaction at Mo and Se edges of nano-flakes. Void spaces between randomly placed sheet enhances the penetration activity of water molecules. The random size and structure of nano-flakes exhibits a high density/concentration of Mo and Se atoms on the edges. This further facilitates the hydrogen bonding with Mo and Se atoms. With increase in humidity levels in the ambience, the bonding ratios increase and the sensing layer impedance decreases accordingly. Weaker bond energy also allows an easier reverse reaction with decrease in humidity levels.

**
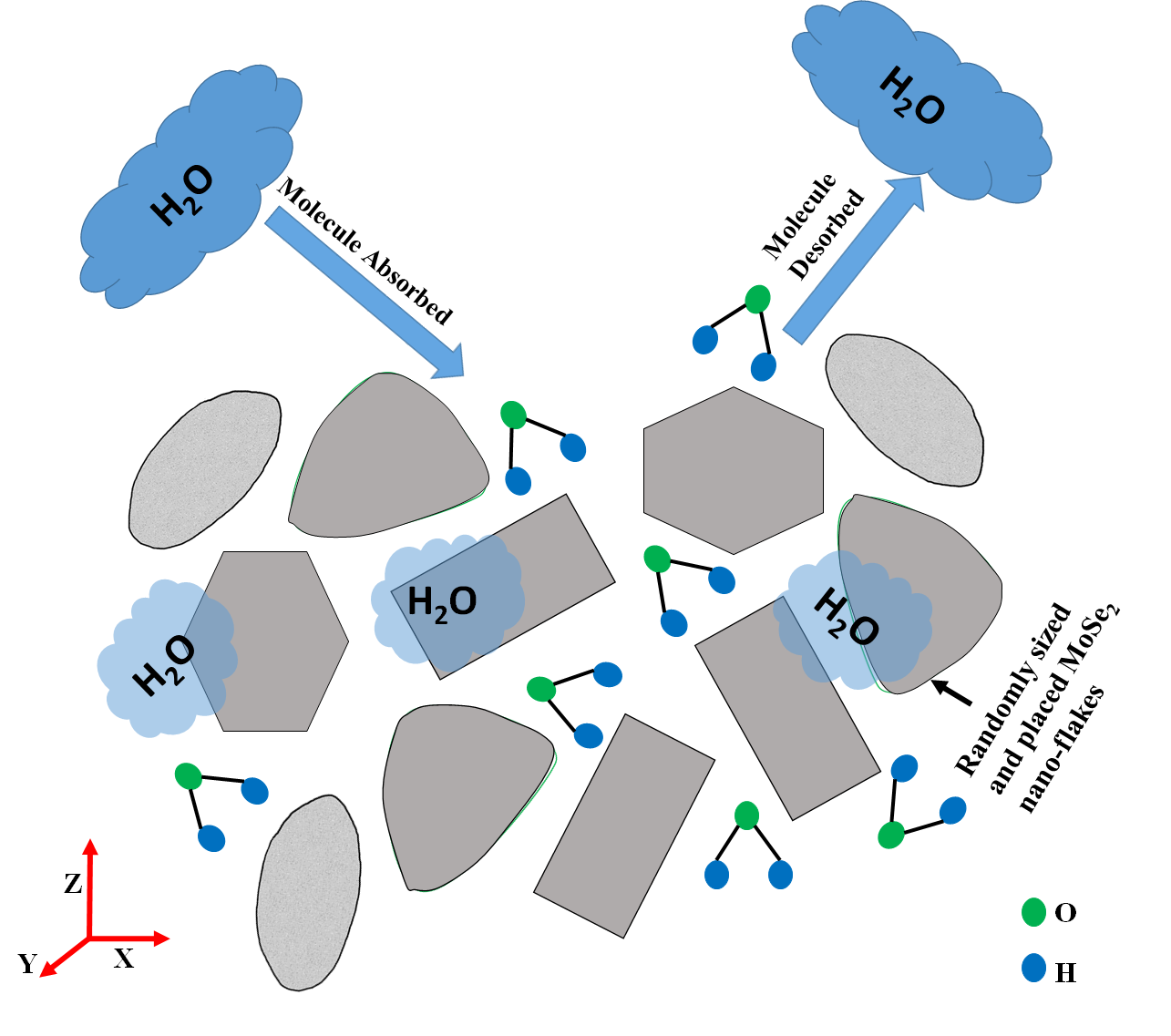
**

**Figure S15. Cross-sectional view of nano-flakes**

**Raman Shift of MoSe_2_**

The Raman shift of MoSe_2_ nano flakes was performed with Lab Ram HR Evolution Raman spectrometer (Horiba Jobin Yvon, France) with excitation source of Ar+ ion laser of wavelength 514 nm, which operates at a power of 10 mW as shown in **Figure S16**. In the Raman spectra, of MoSe2 nano flakes two characteristic peaks at 238.79 cm^−1^ and 286.98 cm^−1^ as shown in Figure S16. The 238.79 cm^-1^ peak corresponds to A_1g_ mode and 286.98 cm^-1^ peak belongs to in-plane E^2^_g1_ mode of MoSe_2_ nano flakes[^8^](#_ENREF_8).


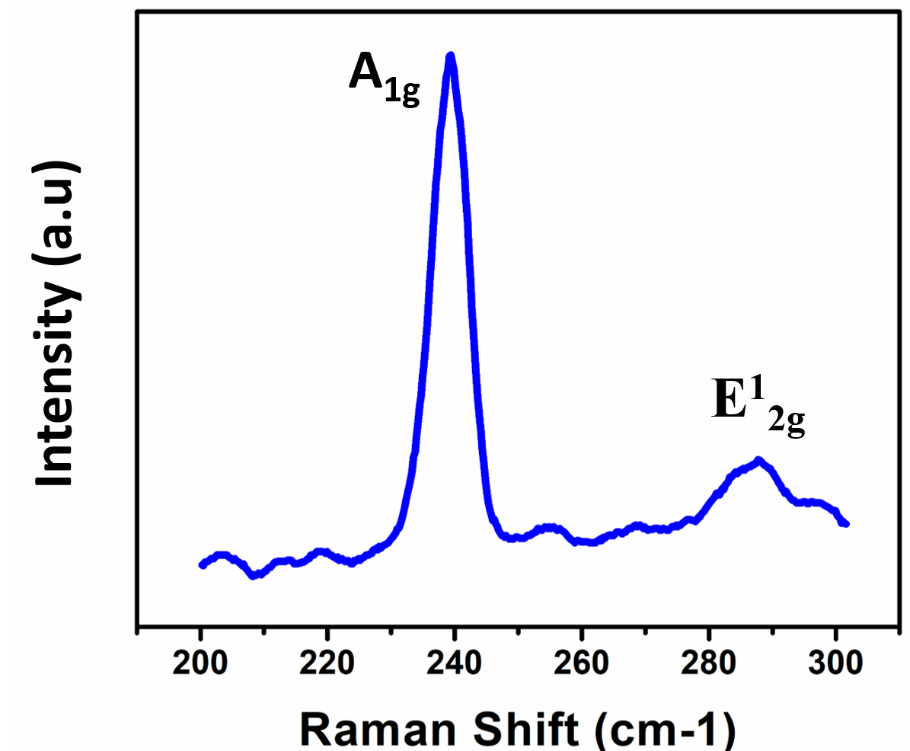


**Figure S16. Raman shift of MoSe_2_ nano-flakes.**

**Electrical read using data acquisition circuit (DAQ)**

The direct electrical read out is performed using data acquisition circuit (DAQ) and oscilloscope, in which fixed resistor (R_fixed_) 2.6 MΩ connected in series with proposed humidity sensor (H_S_). The V_DC_ of 5 Volts was applied across the series connection of R_fixed_ and H_S_. The output was taken across the H_S_ at V_read_ as shown in **Figure S17(a)**. The voltage drop across the H_S_ varies by changing humidity level. To measure the voltage variation across proposed sensor, an Agilent Technologies DSO7052B Digital storage oscilloscope was connected across the H_S_. The sensor was exposed to the human breath as shown in **Figure S17(b)**. The resistance of the H_S_ decreases during exhaling and increased during inhaling. The resistance varies from 5.2 MΩ to 0.25 MΩ from 30% RH (room humidity level) to approximately 100% RH, respectively against human breath (See the detailed explanation in Supplementary Video_human breath movie file.).

**
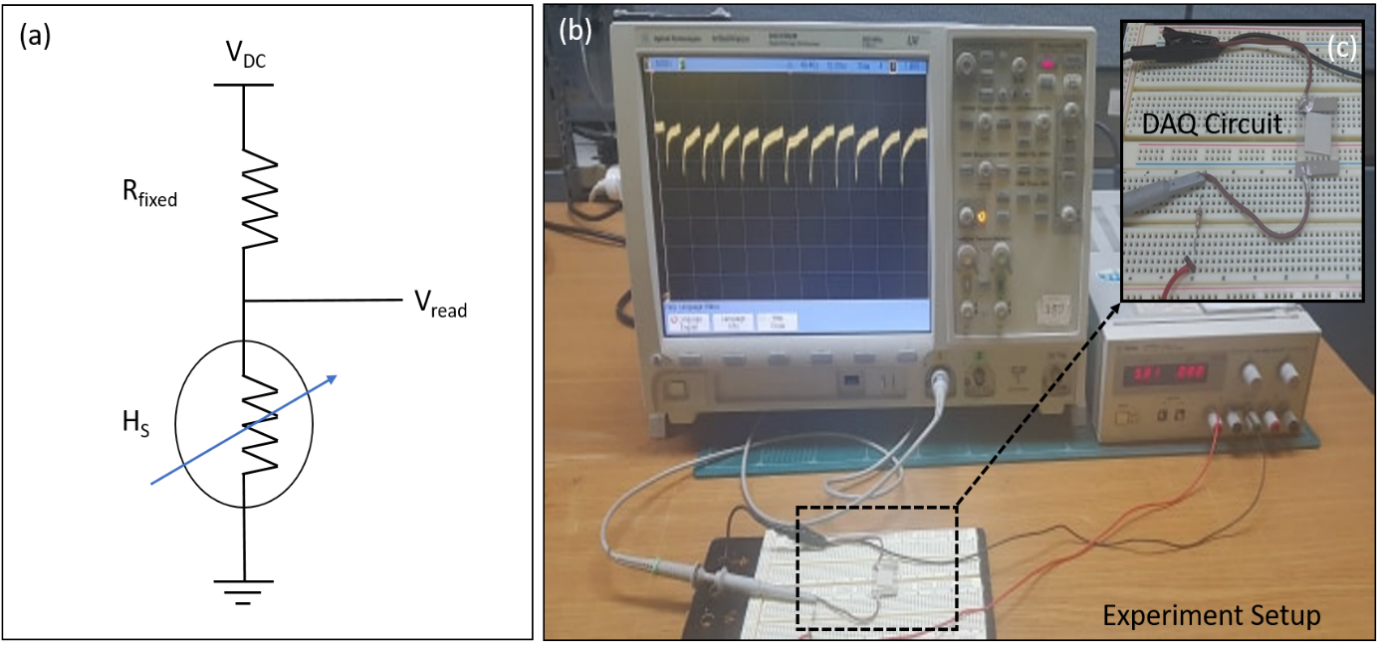
**

**Figure S17.** (a) Sensor interfacing circuit to get a digital data. (b) The experimental setup and the measured photo.

**References**

1 Pemberton, S. A. *A novel approach to multiphysics modeling of heat and mass transfer in porous media* Master of Science thesis, University of Tennessee, Knoxville, (2013).

2 Gibson, P. W. & Charmchi, M. Modeling convection/diffusion processes in porous textiles with inclusion of humidity-dependent air permeability. *International Communications in Heat and Mass Transfer* **24**, 709-724, doi:https://doi.org/10.1016/S0735-1933(97)00056-0 (1997).

3 *Diffusion Equation*, <https://[www.comsol.com/multiphysics/diffusion-equation](http://www.comsol.com/multiphysics/diffusion-equation)> (2015).

4 *Diffusion Coefficient*, <https://[www.comsol.com/multiphysics/diffusion-coefficient](http://www.comsol.com/multiphysics/diffusion-coefficient)> (2017).

5 *Electrostatics, Theory*, <https://[www.comsol.com/multiphysics/electrostatics-theory](http://www.comsol.com/multiphysics/electrostatics-theory)> (2019).

6 Rahman, M. S. A., Mukhopadhyay, S. C. & Yu, P.-L. in *Novel Sensors for Food Inspection: Modelling, Fabrication and Experimentation* 11-35 (Springer, 2014).

7 Riaz, K., Iqbal, A., Mian, M. U. & Bazaz, S. A. Active gap reduction in comb drive of three axes capacitive micro accelerometer for enhancing sense capacitance and sensitivity. *Microsystem Technologies* **21**, 1301-1312 (2015).

8 Samikannu, S. & Sivaraj, S. Dissipative soliton generation in an all-normal dispersion ytterbium-doped fiber laser using few-layer molybdenum diselenide as a saturable absorber. *Optical Engineering* **55**, 081311, doi:https://doi.org/10.1117/1.OE.55.8.081311 (2016).
